# Spatiotemporal Dynamics of Cerebral Vascular Permeability in Type 2 Diabetes-Related Cerebral Microangiopathy

**DOI:** 10.3389/fendo.2021.805637

**Published:** 2022-01-11

**Authors:** Ying-Chen Chen, Bing-Ze Lu, Yu-Chen Shu, Yuan-Ting Sun

**Affiliations:** ^1^ Department of Medical Imaging, National Cheng Kung University Hospital, College of Medicine, National Cheng Kung University, Tainan, Taiwan; ^2^ Department of Radiology, Kaohsiung Municipal United Hospital, Kaohsiung, Taiwan; ^3^ Department of Mathematics, College of Science, National Cheng Kung University, Tainan, Taiwan; ^4^ Department of Neurology, National Cheng Kung University Hospital, College of Medicine, National Cheng Kung University, Tainan, Taiwan; ^5^ Department of Medical Genomics, National Cheng Kung University Hospital, College of Medicine, National Cheng Kung University, Tainan, Taiwan

**Keywords:** diabetes mellitus, blood–brain barrier, cerebral small vessel disease, permeability, microangiopathy

## Abstract

**Aims:**

Diabetes-related cerebral microangiopathy can manifest as cerebral small vessel disease (CSVD) and exhibit cognitive decline. To find the early change of function in advance, this study examined the spatiotemporal dynamics of cerebral vascular permeability (*Ktrans*) in the progression of type 2 diabetes mellitus (T2DM).

**Methods:**

*Ktrans* was cross-sectionally measured in T2DM and non-diabetes groups with or without CSVD using dynamic contrast-enhanced MRI (DCE-MRI).

**Results:**

In all patients with T2DM, the *Ktrans* of white matter (WM) was increased, whereas the *Ktrans* of gray matter (GM) was increased only in T2DM with CSVD. The involvement of WM was earlier than GM and was before the CSVD features could be visualized on MRI. Among the commonly available four CSVD items of MRI, microbleeds were the most sensitive, indicating the increased permeability in all patients. Increased *Ktrans* in T2DM was more associated with moderate WM hyperintensity but less with the presence of lacunae or multiple perivascular spaces, in contrast to patients without diabetes. The differential correlation suggested distinct mechanisms underlying diabetes-related CSVD and other CSVDs.

**Conclusions:**

This study highlights the early development of cerebral microangiopathy with increased BBB leakage in T2DM, before the CSVD features can be visualized on MRI. The results may increase the proactivity of clinicians in recognizing the subsequent neurological comorbidities.

## Introduction

Cerebral small vessel disease (CSVD) represents a heterogeneous group of disorders. The hypothetical etiologies include microatheroma, endothelial dysfunction, inflammation, and altered microvascular blood–brain barrier (BBB) integrity (1). Type 2 diabetes mellitus (T2DM) is highly associated with CSVD (2–4) that can manifest as symptomatic or silent lacunar infarcts and cognitive decline. T2DM-related CSVD can be diabetic microangiopathy involving the brain. Given the increasing global prevalence of diabetes, CSVD and the accompanying neurological disability are considerable challenges for healthcare systems (5) and thus are becoming increasingly crucial (4–7).

Vascular permeability was recently reported as a critical mechanism underlying dementia (8). Diabetes triggers various vascular pathologies, including increased vascular permeability that may contribute to cognitive decline and other morbidities such as ischemic stroke (9, 10). Therefore, the increase in BBB permeability in patients with T2DM might be an early tissue-level dysfunction associated with subsequent cognitive decline.

This study hypothesized that the increase in vascular permeability would present early in patients with T2DM, even before the development of CSVD features on MRI. This temporal evolution may be unique to T2DM-related CSVD that represents cerebral microangiopathy similar to other target organs in diabetes. To test the hypothesis, we performed dynamic contrast-enhanced MRI (DCE-MRI) to examine the spatiotemporal dynamics of cerebral vascular permeability (11) in T2DM patients with or without CSVD. Then, we correlated the CSVD items of conventional MRI with cerebral permeability and compared the findings with CSVD patients without diabetes to identify the unique features of the T2DM-specific cerebral microangiopathy in a heterogeneous CSVD group.

## Subjects, Materials, and Methods

### Study Design

This study was carried out at the National Cheng Kung University Hospital (Tainan, Taiwan) and Kaohsiung Municipal United Hospital (Kaohsiung, Taiwan). The local Institutional Review Board approved the protocol (Approval No. A-BR-106-081). Four groups of subjects, T2DM without CSVD, T2DM with CSVD, patients with CSVD but without T2DM, and controls (individuals without T2DM, without CSVD), were recruited from the neurology outpatient clinic in National Cheng Kung University Hospital. Demographic data, permeability, and CSVD scores on MRI between the four groups were analyzed cross-sectionally.

### Participants

In recruiting subjects, the diagnosis of T2DM, followed by the American Diabetes Association (12–14), should exist on medical records for at least 5 years. The stages of chronic kidney disease (CKD) followed the guidance from the National Kidney Foundation (15). Hypertension, if it exists, should be well controlled (16). The presence of CSVD was defined as a total CSVD score of >1 on MRI (17). Patients with intracranial tumor, cerebral venous thrombosis, hemorrhagic stroke, immune-mediated neuroinflammatory disease, hydrocephalus, >50% stenosis in any large intracranial vessel, hereditary neurodegenerative disorder, advanced CKD (>stage III), liver cirrhosis, history of posterior reversible encephalopathy syndrome, and acute infection were ineligible for this study. Demographic data, dementia, hypertension, hyperlipidemia, levels of HbA1c, total cholesterol, low-density lipoprotein cholesterol, and creatinine were collected. Patients with a Mini-Mental Status Examination score of ≤26 or a Clinical Dementia Rating score of ≥0.5 were considered to have dementia.

### Imaging Protocol

#### Structural Imaging

All participants underwent conventional structural imaging and then DCE-MRI on an MRI system (Ingenia 3.0 T, Philips Healthcare, the Netherlands). Conventional structural sequences contained axial T1-weighted images, axial T2-weighted fat-saturation images, and axial T2-weighted fluid-attenuated inversion recovery (FLAIR) images, as well as susceptibility-weighted images to evaluate microbleeding.

#### DCE-MRI

**Figure 1:**
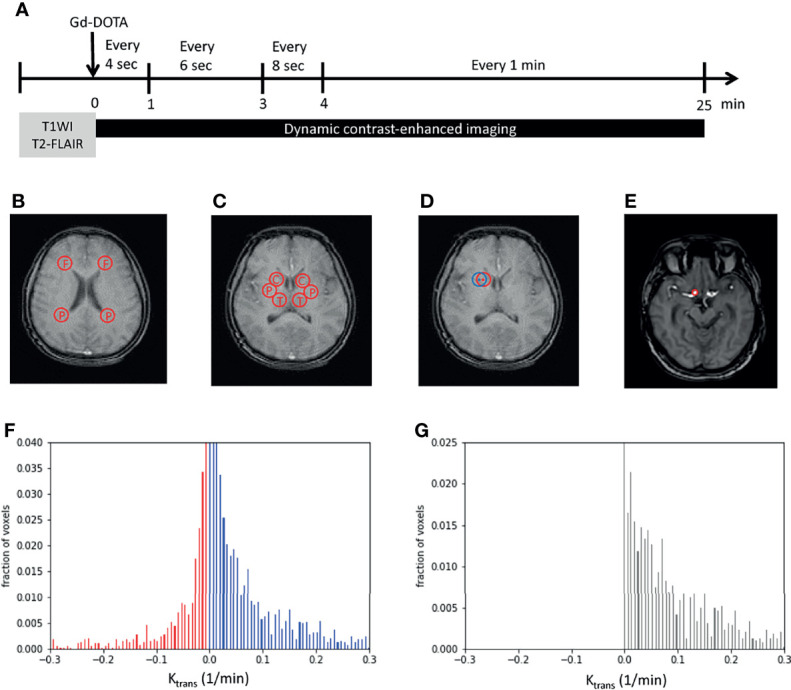
Image processing. **(A)** Imaging protocols included structural images (T1WI and T2-FLAIR images) and dynamic imaging. The time bar shows dynamic imaging intervals during the 25-min acquisition time. **(B, C)** Examples show the two regions of interest (ROIs) in the white matter (WM) **(B)**, namely, the frontal and parietal WM, and three ROIs in the gray matter **(C)**, namely, the caudate head, putamen, and thalamus. **(D)** An example shows the perturbation of ROIs. The center and radius of the initial ROI (in red) randomly oscillated within three pixels to obtain another ROI (in blue) that was near to the initially examined ROI (in red). **(E)** The internal carotid artery was selected and set as the region of plasma in Patlak analysis. **(F, G)** The *K_trans_* of each pixel was plotted as a histogram. Red bars indicated negative *K_trans_* values and were considered as noise **(F)**. The noise was supposed to exist with the same distribution on the positive side. By subtracting noise (red bars) from the original histogram (blue bars), tissue permeability was observed (black bars in **G**). *x*-axis, *K_trans_*; *y*-axis, the fraction of voxels. F, frontal; P, parietal; C, caudate head; P, putamen; T, thalamus; Gd-DOTA, gadoterate meglumine; T1WI, T1-weighted image; T2-FLAIR, T2-weighted fluid-attenuated inversion recovery.

To examine vascular permeability on MRI, two reference series using axial T1-weighted fast-field echo (T1-FFE) with flip angles of 5° and 15° were initially performed. Subsequently, DCE-MRI of the whole brain was performed using axial T1-FFE with a flip angle of 8° (repetition time/echo time, 2.8/1.32 ms; slice width, 3 mm; no slice gap; field of view, 230 × 202 × 108 mm^3^; matrix, 116 × 115). An intravenous bolus injection of a gadolinium-based contrast agent, gadoterate meglumine (Gd-DOTA), was administered after the first imaging of this series at a dose of 0.1 mmol/kg. The DCE-MRI sequence was repeated 63 times over a 25-min acquisition period. The details of imaging intervals are as follows: an imaging interval of 4 s during a postcontrast injection of 0–1 min, an imaging interval of 6 s during a postcontrast injection of 1–3 min, an imaging interval of 8 s during a postcontrast injection of 3–4 min, and an imaging interval of 1 min during a postcontrast injection of 4–25 min (**Figure 1A**).

### Image Analysis

To overcome the subtle inhomogeneity between T1 images, we used the intensity of the fat tissue near the constant brightness of each image as a reference for the following calibration. The averaged intensity of the three regions of fat around the eyes was adjusted to a value of 1 in each image. Five regions of interest (ROIs) were chosen: frontal WM, parietal WM, caudate head (CH), putamen, and thalamus. The frontal and parietal WM measurements were obtained using an axial view image at the level above the CH. The centers of the ROIs were laterally next to the frontal and parietal horns of the lateral ventricles (**Figure 1B**). The CH, putamen, and thalamus measurements were obtained using an axial view image at the central thalamus and Sylvian fissure (**Figure 1C**). To avoid bias caused by the inhomogeneity of the selected ROIs, the center and radius of each ROI were randomly oscillated within 3 pixels to obtain six ROIs close to the chosen ROI (**Figure 1D**) initially. The intensities of the six corresponding oscillated regions were averaged to represent the selected ROI initially.

### Determination of BBB Permeability (*K_trans_*)

A Patlak graphical analysis technique was adopted to determine pixel-wise **BBB permeability** (*K_trans_*) in an ROI under the assumption of the irreversible uptake pharmacokinetics of Gd-DOTA (18). In a compartment model, the relative concentration *C*(***r***, *t*) was modeled in terms of the contrast of the initial intensity as follows:


$$C\left(r,t\right)=-\frac{K}{T_{e}}\ln\left(\frac{S\left(r,t\right)}{S\left(r,0\right)}\right)$$

where *T_e_* is the echo time, K is the concentration constant, and *S*(***r***, *t*) is the intensity of the pixel location ***r*** at time *t* after Gd-DOTA injection. The concentration *C_i_* (***r***, *t*) in the ROI was estimated using the following equation:


$$C_{i}\left(r,t\right)=K_{trans}\left(r\right)\int_{0}^{t}C_{p}\left(\tau \right)d\tau +V(r)\;•\;C_{p}\left(t\right),$$

where *V*(***r***) is the distribution of Gd-DOTA in the vessels, *C_p_* (*t*) is the concentration of Gd-DOTA in the plasma, and *K_trans_* (***r***) is the rate at which Gd-DOTA enters the brain parenchyma, indicating permeability. We obtained the plasma Gd-DOTA intensity by marking the internal carotid artery as the region of plasma (ROP; **Figure 1E**). In addition, we perturbed the selected ROP to reduce the inhomogeneity between pixel values. The averaged relative concentration of ROP was calculated using the following equation:


$$C_{p}\left(t\right)=\int \int_{r\in ROP}^{}C\left(r,t\right)dA/\int \int_{r\in ROP}^{}dA$$

### Noise Elimination

To minimize noise produced by imaging procedures, a noise elimination method that involved plotting the histogram of *K_trans_* in an ROI was applied (19). After all the *K_trans_* values of each pixel were computed, normalization preprocessing was performed to obtain the pixel-wise proportion of each *K_trans_*. The cumulative sum of all *K_trans_* values was set as 1. Subsequently, all *K_trans_* values were plotted pixel-wise on a histogram, with the proportion of the pixel in an ROI presented on the *y*-axis (**Figure 1F**). Because *K_trans_* in the model refers to the rate of entry into an irreversible compartment, a negative value representing an opposite direction of the movement of Gd-DOTA from the parenchyma to the vessel was considered noise (red bars in **Figure 1F**). In addition, the noise was supposed to be present on the positive part of the histogram with a mirror distribution of the negative part. After subtracting the estimated noise (red bars, **Figure 1F**) from the positive *K_trans_* histogram (blue bars, **Figure 1F**), the remaining cumulative sum of the bars was identified as the approximate *K_trans_* in an ROI (**Figure 1G**).

### CSVD Scoring

The presence of CSVD was determined by viewing structural MRI images. The CSVD score was obtained by summating the four parameters raised by Staals et al. (17), which had been applied and validated in multiple fields of neurology (20–22): lacunes, microbleeds, perivascular space (PVS), and grading of periventricular leukoaraiosis, namely, WM hyperintensity (WMH) (17). The WMH was graded from 0 to 3 using the Fazekas scale. Patients were considered to have CSVD if their CSVD score was >1.

### Software and Statistical Analysis

The total *K_trans_* represented an average from *K_trans_* of WM and *K_tran_*_s_ of GM. *K_trans_* of WM was an average from *K_trans_* of frontal and parietal WM. *K_trans_* of GM was an average from *K_trans_* of CH, putamen, and thalamus. The imaging processing and scientific computing of *K_trans_* were performed using MATLAB (2019b, The MathWorks, Natick, Massachusetts, USA) and the Python programming language (version 3.7). Statistical analyses were performed using Prism (version 6; GraphPad Software, La Jolla, CA, USA) and the R programming language (version 4.0.4). Unpaired Student’s *t*-test, Mann–Whitney *U* test, Fisher’s exact test, one-way analysis of variance (ANOVA), or Kruskal–Wallis test were used according to data type and group number. Normality tests were conducted for continuous data before comparisons. To test the correlation, Kendall’s tau was used for ordinal data, and a permutation-based linear model with 10,000 random shufflings was used for continuous numerical data. Significance was set at *p* < 0.05.

## Results

Thirty-one patients with T2DM and 14 individuals without diabetes were enrolled. Seven were excluded because of numerous motion artifacts on their MRI. Finally, 25 patients with T2DM (11 women, 49–88 years old) and 12 individuals without T2DM (8 women, 21–81 years old) were included in the analysis. The demographic data of the excluded subjects were not different from the study subjects. Patients with T2DM were further divided into two groups based on the MRI-based CSVD score: T2DM without CSVD (*n* = 12, 7 female, 49–78 years old) and T2DM with CSVD (*n* = 13, 4 female, 63–88 years old) (**Table 1**). The distribution of sex and the prevalence of hypertension did not differ among the three groups (*p* = 0.25 and 0.18, respectively, Fisher’s exact test). Subjects of T2DM with CSVD were older (*p* = 0.004, one-way ANOVA with the multiple comparisons test), had a higher proportion of dementia (*p* = 0.034, Fisher’s exact test), and had a more advanced stage of CKD (*p* = 0.036, Kruskal–Wallis test). Among patients with T2DM, the HbA1c level was equal (*p* = 0.22, Student’s *t*-test) between the CSVD and non-CSVD groups. The scores of individual CSVD items and the total CSVD score did not differ between the control and T2DM without CSVD groups (total score, *p* = 0.301; lacune, *p* = 0.9; PVS, *p* = 0.39; WMH, *p* = 0.62; microbleeds, *p* = 0.195; Mann–Whitney *U* test).

**Table 1:** Demographic data of patients with and without diabetes.

	Total (*N* = 37)	Diabetes (*N* = 25)		Non-diabetes (*N* = 12)	*p*-value
		CSVD (*N* = 13)	No CSVD (*N* = 12)		
Gender (Female,%)	19 (51.4)	4 (30.7)	7 (58.3)	8 (66.7)	0.257
Age (mean ± SD)	64.83 ± 15.26	74.61 ± 8.75	63.25 ± 10.4	55.83 ± 19.32	0.004**
Hypertension (*N*, %)	20 (54.1)	8 (61.5)	8 (66.7)	5 (33.3)	0.313
Dementia (*N*, %)	16 (43.2)	10 (76.9)	6 (50)	3 (25)	0.034*
CKD stage (median, [IQR])	1 [1,2]	2 [1.5,3]	1 [1,2]	1 [1,1]	0.036*

DM, diabetes mellitus; CSVD, cerebral small vessel disease; CKD, chronic kidney disease; SD, standard deviation; *p < 0.05; **p < 0.01.

### Temporal Dynamics: T2DM Showed an Impact on the Increase in *K_trans_* in Addition to the Effect of Aging, Particularly in Diabetes With CSVD Group

**Figure 2:**
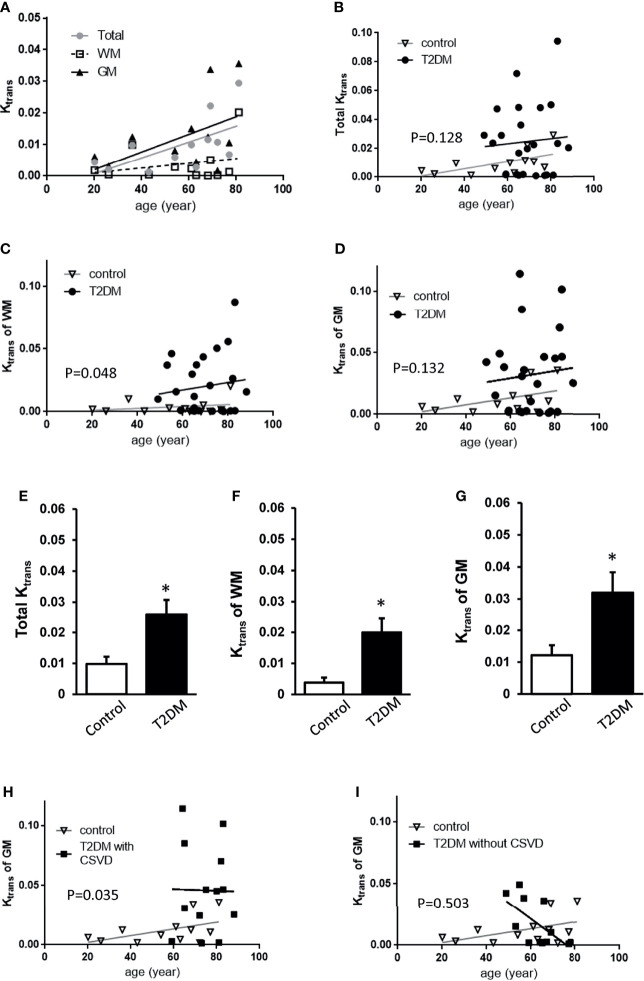
Comparisons of *K_trans_* among control, T2DM, and T2DM with CSVD groups. **(A)** The averaged total *K_trans_*, *K_trans_* of the WM, and GM plotted against age in the control group (*N* = 12). **(B–D)** The total *K_trans_* **(B)**, *K_trans_* of the WM **(C)**, and GM **(D)** plotted against age in T2DM (*N* = 25) and control groups (*N* = 12). **(E–G)** Comparisons of total *K_trans_* **(E)**, *K_trans_* of the white matter (WM) **(F)**, and *K_trans_* of the gray matter (GM) **(G)** between patients with T2DM and controls. Unpaired *t*-test. *p < 0.05. **(H, I)** The *K_trans_* of the GM plotted against age in T2DM with CSVD and the control group **(H)** and in T2DM without CSVD and the control group **(I)**. A permutation-based linear model with 10,000 random shufflings. *x*-axis, age; *y*-axis, *K_trans_*. WM, white matter; GM, gray matter; T2DM, type 2 diabetes mellitus; CSVD, cerebral small vessel disease.

In all subjects, the total *K_trans_*, *K_trans_* of WM, and *K_trans_* of GM all increased with age (**Figures 2A–D**), approximately following the equation of total *K_trans_* = (0.2517 * age − 4.254)/1000 (*R* ^2^ = 0.3638, *p* = 0.0379) in the control group and total *K_trans_* = [0.1768 * age + 12.73]/1,000 in the T2DM group. Before the adjustment for age, the T2DM group had increased total *K_trans_*, *K_trans_* of WM, and *K_trans_* of GM (*p* = 0.0258, 0.0199, and 0.0319, respectively, Student’s *t*-test, **Figures 2E–G**). In order to adjust the effect of aging, a permutation-based linear model with 10,000 random shufflings of patients and healthy controls was performed to test the correlation. A significant effect of diabetes on the *K_trans_* of WM (*p* = 0.048, **Figure 2F**) but not on total *K_trans_* and *K_trans_* of GM was found (*p* = 0.128, *p* = 0.132, respectively, **Figures 2E–G**) between T2DM and the control group.

**Figure 3:**
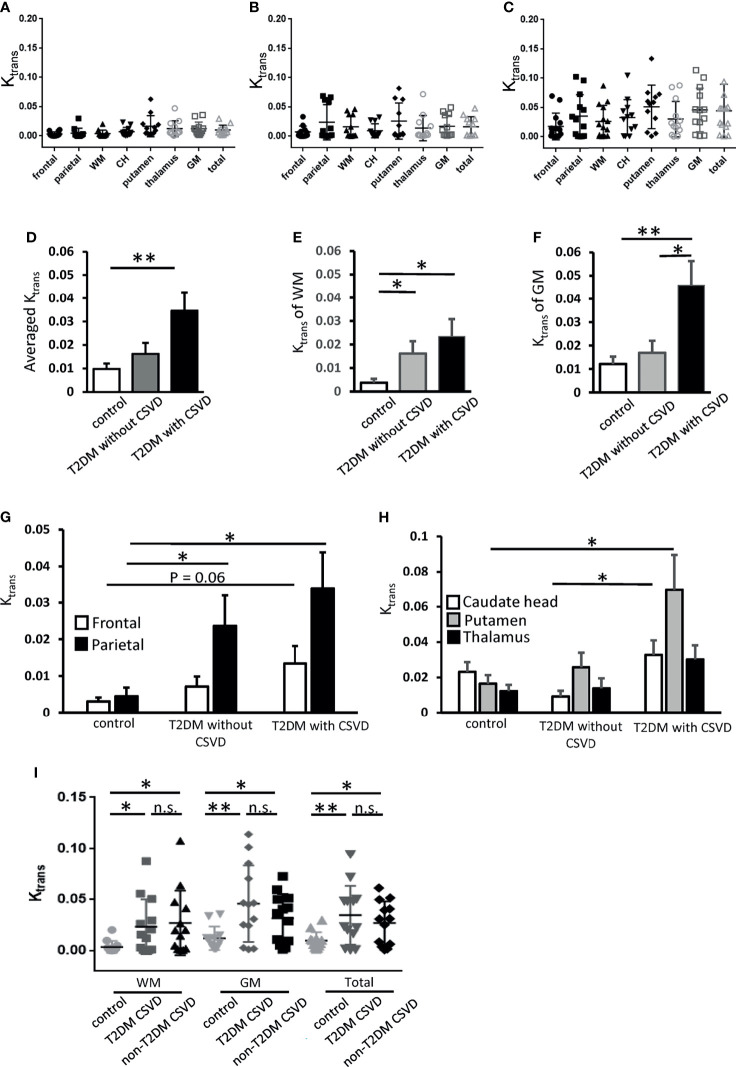
*K_trans_* of each cerebral area in the control, T2DM, and T2DM with CSVD groups. **(A–C)** *K_trans_* of each area in the control (*N* = 12) **(A)**, T2DM without CSVD (*N* = 12) **(B)**, and T2DM with CSVD groups (*N* = 13) **(C)**. **(D–F)** Comparisons of the averaged total *K_trans_* **(D)**, *K_trans_* of the WM **(E)**, and *K_trans_* of the GM **(F)** among control, T2DM without CSVD, and T2DM with CSVD groups. **(G, H)** Comparisons of *K_trans_* in the frontal and parietal WM **(G)** and *K_trans_* in the caudate head (CH), putamen, and thalamus **(H)** between control, T2DM without CSVD, and T2DM with CSVD groups. **(I)** Comparisons of *K_trans_* in the WM, GM, and total *K_trans_* between the control group, CSVD patients with T2DM, and CSVD patients without T2DM. Unpaired *t*-test. NS, no significance; *p < 0.05; **p < 0.01. WM, white matter; GM, gray matter; CH, caudate head; T2DM, type 2 diabetes mellitus; CSVD, cerebral small vessel disease.

Based on the assumption that CSVD features on MRI represented a more advanced stage of T2DM than no CSVD features on MRI (23), T2DM patients were further divided into T2DM with CSVD and T2DM without CSVD. The effect of diabetes on *K_trans_* of GM after age adjustment was shown in the T2DM with CSVD group (*p* = 0.035, permutation test for 10,000 random resamples, **Figure 2H**) but not in the T2DM without CSVD group (*p* = 0.503, permutation test for 10,000 random resamples, **Figure 2I**). **Figures 3A–C** show the *K_trans_* of each cerebral area in the control (A), T2DM without CSVD (B), and T2DM with CSVD (C) groups. The increase of *K_trans_* was found in every ROI in the T2DM with CSVD group (**Figure 3C**). The higher averaged total *K_trans_* of T2DM patients shown in **Figure 2E** was mainly contributed by T2DM patients with CSVD (*p* = 0.008, Student’s *t*-test, **Figure 3D**). No statistical difference was found between control and T2DM without CSVD, or between T2DM with or without CSVD (*p* = 0.24 and 0.093, respectively, Student’s *t*-test, **Figure 3D**).

### Spatial Dynamics: *K_trans_* of WM Was Increased in All Patients With T2DM, Whereas *K_trans_* of GM Was Increased Only in Patients With T2DM and CSVD

To examine the spatial dynamics of *K_trans_* in T2DM, the *K_trans_* values of WM and GM were analyzed separately. The *K_trans_* of the WM was increased in all patients with T2DM, irrespective of the absence or presence of CSVD (*p* = 0.04 and 0.02, respectively, Student’s *t*-test, **Figure 3E**). The *K_trans_* of the GM was only increased in the T2DM with CSVD group that had an advanced diabetes status (*p* = 0.006, Student’s *t*-test, **Figure 3F**). In addition, among all patients with T2DM, the *K_trans_* of the GM was higher in the T2DM with CSVD group than in the T2DM without CSVD group (*p* = 0.023, Student’s *t*-test, **Figure 3F**).

Regarding the WM, parietal region showed an increase in *K_trans_* in all patients with T2DM (T2DM without CSVD vs. control, *p* = 0.04; T2DM with CSVD vs. control, *p* = 0.016; Student’s *t*-test, **Figure 3G**), whereas frontal WM only showed a marginal statistical significance (*p* = 0.06, Student’s *t*-test, **Figure 3G**). Regarding the GM, the *K_trans_* of the putamen and CH were higher in the T2DM with CSVD group (T2DM with CSVD vs. control, *p* = 0.027; T2DM with CSVD vs. T2DM without CSVD, *p* = 0.021, Student’s *t*-test, **Figure 3H**). In summary, in the T2DM group, the increased *K_trans_* of the WM was most prominent in the parietal area, and the increased *K_trans_* of the GM was mainly found in the CH and putamen.

### *K_trans_* Was Not Correlated With the HbA1c Level

The HbA1c level, an averaged status of recent glycemic control, in patients who underwent DCE-MRI was not correlated with the *K_trans_* of the WM or GM (*r* ^2^ = 0.0157 and 0.0176, respectively, Pearson’s correlation). Even analyzing T2DM without CSVD and T2DM with CSVD groups separately, the correlation between the HbA1c level and *K_trans_* was not observed. We further divided patients with T2DM according to their recent glycemic control into two groups, HbA1c < 8 and HbA1c ≥ 8. The *K_trans_* of the WM or GM did not significantly differ between the two groups (*p* = 0.235 and 0.173, respectively, Student’s *t*-test).

### High CSVD Score Correlated With the Increased *K_trans_* in All Patients With CSVD

We further clarify whether the increase in *K_trans_* generally presents in all CSVDs or is a specific feature for T2DM-related CSVD. Nondiabetic patients with CSVD were enrolled (*n* = 13 [7 men and six women], aged 74.9 ± 11 years). Their sex, age, the prevalence of hypertension or dementia, the stage of CKD, the total CSVD score, the scores of individual CSVD items, and the *K_trans_* values of the five ROIs did not differ from the T2DM patients with CSVD (**Table 2**). In both T2DM CSVD and non-diabetes CSVD groups, their *K_trans_* of WM, GM, and total *K_trans_* were higher than the control group (**Figure 3I**), suggesting the increase in *K_trans_* present in all CSVDs.

**Table 2:** Comparisons of demographic data between patients with CSVD with and without T2DM.

	T2DM with CSVD	Non-diabetes CSVD	*p*-value
	(*N* = 13)	(*N* = 12)	
Gender (Female) (*N*, %)	4, 30.7	9, 75	0.165
Age (mean ± SD)	68.78 ± 10.45	74.9 ± 11.06	0.94
Hypertension (*N*, %)	8, 61.5	7, 58.3	0.11
Dementia (*N*, %)	10, 76.9	9, 75	0.57
CKD stage (median, [IQR])	2 [1.5,3]	2 [1,2]	0.31
Total CSVD score	3 [2.5,3]	3 [2,5]	0.301
PVS score	0 [0,1]	1 [0,1]	0.393
Lacune score	1 [0,1]	1 [0,1]	0.99
WMH score	1 [1,1]	1 [1,1]	0.627
MB score	1 [0.5,1]	1 [1,2]	0.195
Total *K_trans_*	0.035 ± 0.028	0.027 ± 0.02	0.449
WM *K_trans_*	0.023 ± 0.025	0.027 ± 0.03	0.756
Frontal WM *K_trans_*	0.013 ± 0.017	0.0194 ± 0.0197	0.431
Parietal WM *K_trans_*	0.033 ± 0.036	0.0247 ± 0.0221	0.474
GM *K_trans_*	0.046 ± 0.036	0.032 ± 0.022	0.256
CH *K_trans_*	0.032 ± 0.029	0.023 ± 0.02	0.365
Putamen *K_trans_*	0.069 ± 0.072	0.045 ± 0.032	0.219
Thalamus *K_trans_*	0.03 ± 0.029	0.027 ± 0.02	0.803

CKD, chronic kidney disease; SD, standard deviation; CSVD, cerebral small vessel disease; PVS, perivascular space; WMH, white matter hyperintensity; MB, microbleed; WM, white matter; GM, gray matter; CH, caudate head; T2DM, type 2 diabetes.

### The Increased *K_trans_* in T2DM and Non-T2DM CSVD Correlated With Distinct CSVD Items

Because the DCE-MRI protocol is time-consuming, it may not be routinely used in conventional medical practice. In addition, DCE-MRI is highly dependent on patients’ cooperation and is challenging to perform in patients with moderate to severe dementia. To improve the accessibility and applicability of the estimation of BBB permeability, we utilized the individual items of MRI CSVD scores, which are more generally available, to indicate the increase in *K_trans_*.

Initially, in recruiting subjects, CSVD was defined as MRI CSVD score >1. Any of the 4 CSVD items can contribute a positive score. However, the correlation between an individual item and *K_trans_* was low and varied (Kendall’s tau between total *K_trans_* and lacune numbers, total *K_trans_* and PVS numbers, 0.218 and −0.22, respectively). Thus, we tried to find better combinations of each MRI CSVD item with different thresholds to indicate *K_trans_*.

For patients with T2DM, all of them were reclassified according to the various cutoff points of each CSVD item. For instance, patients with T2DM were reclassified into two groups, according to WMH < 1 and WMH ≥ 1. Subsequently, the *K_trans_* values of the newly generated two groups were compared. For the four CSVD items, WMH values were assigned four grades (WMH = 0, 1, 2, or 3). The PVS ranged from 0 to >20, microbleeds ranged from 0 to >15, and lacunes ranged from 0 to 5. The combinations of the aforementioned items with various cutoff values generated 4,500 classification criteria, including the criterion of a single item and the criteria of ≥2 combined items. Among 4,500 criteria, any one generated two groups with extremely uneven sample sizes (*n* < 8 in one group) was discarded. Finally, 76 classification criteria remained. After dividing patients with T2DM using any of the 76 criteria, and comparing the *K_trans_* of the five ROIs between two groups, finally only six criteria generated two groups of statistically different *K_trans_*.

Among the four MRI features of CSVD, the presence of microbleeds was the most sensitive indicator for a significantly increased *K_trans_* in multiple brain regions, including the CH, putamen, and parietal WM (**Table 3**). The addition of other CSVD items to the microbleeds, including PVS ≥ 2 and WMH ≥ 2, did not change the statistical result. In patients with no microbleeds on MRI, WMH ≥ 2 alone showed marginal significance in suggesting an increased *K_trans_* in the putamen and parietal WM (*p* = 0.065 and *p* = 0.055, respectively). The presence of the PVS or lacune alone did not indicate an increased *K_trans_* in patients with T2DM.

**Table 3:** Statistical power of various combinations of CSVD items in determining *K_trans_* among patients with T2DM.

Criteria	Sensitive region	*p*-value
MB ≥ 1	CH	0.044*
	Putamen	0.047*
	Parietal WM	0.04*
MB ≥ 1 and WMH ≥ 1	CH	0.044*
	Parietal WM	0.04*
MB ≥ 1 and PVS ≥ 2	CH	0.044*
	Putamen	0.047*
	Parietal WM	0.04*
MB ≥ 1 and PVS ≥ 3	CH	0.044*
	Putamen	0.047*
	Parietal WM	0.04*
MB ≥ 1 and WMH ≥ 1 and PVS ≥ 2	CH	0.044*
MB ≥ 1, WMH ≥ 1 and PVS ≥ 3	CH	0.044*
WMH ≥ 2	Putamen	0.065
	Parietal WM	0.055
WMH ≥ 2 and PVS ≥ 2	Parietal WM	0.055

SD, standard deviation; CSVD, cerebral small vessel disease; PVS, perivascular space; WMH, white matter hyperintensity; MB, microbleed; WM, white matter; GM, gray matter; CH, caudate head; T2DM, type 2 diabetes. *P < 0.05.

### *K_trans_* Correlated With Distinct CSVD Features in Patients Without T2DM

To test the hypothesis that T2DM-related CSVD may have distinct pathophysiology from non-diabetes CSVD, the aforementioned methods were also applied to patients without diabetes. Similar to T2DM patients, the presence of microbleeds suggested an increase in *K_trans_* in non-T2DM patients with CSVD (**Table 4**). In contrast to the T2DM group, the presence of lacune alone or multiple PVSs (≥6) alone indicated an increase in *K_trans_* in non-T2DM patients with CSVD. Although moderate WMH (≥2) showed marginal significance in indicating *K_trans_* in the T2DM group, it was not an indicator in the non-diabetes group.

**Table 4:** Statistical power of various combinations of CSVD items in determining the *K_trans_* in nondiabetes group.

Criteria	Sensitive region	*p*-value
MB ≥ 1	CH	0.016*
	Frontal WM	0.013*
MB ≥ 2	CH	0.0086**
	Frontal WM	0.01*
MB ≥ 1 and PVS ≥ 4	CH	0.003**
	Frontal WM	0.02*
MB ≥ 2 and PVS ≥ 4	CH	0.0017**
MB ≥ 2 and PVS ≥ 7	Frontal WM	0.027*
MB ≥ 2 and WMH ≥ 2	CH	0.05
	Frontal WM	0.075
Lacune ≥ 1	CH	0.032*
PVS ≥ 6	CH	0.077
	Frontal WM	0.029*
PVS ≥ 6 and WMH ≥ 1	CH	0.047*
	Frontal WM	0.024*
PVS ≥ 7	Frontal WM	0.035*
PVS ≥ 8	Frontal WM	0.028*

CKD, chronic kidney disease; SD, standard deviation; CSVD, cerebral small vessel disease; PVS, perivascular space; WMH, white matter hyperintensity; MB, microbleed; WM, white matter; GM, gray matter; CH, caudate head; F, frontal white matter. *P < 0.05; **P < 0.01.

## Discussion

This study demonstrated the early presence of cerebral microangiopathy in T2DM, with distinct mechanisms from other CSVD. The following evidence supported this conclusion: First, the increased *K_trans_* in WM was prior to the presence of visible CSVD features on MRI. Second, the increased *K_trans_* in GM developed in advanced T2DM. Third, the increased *K_trans_* was more associated with the long-lasting progress of T2DM, less with the recent glycemic control. Fourth, among the four CSVD features on MRI, the presence of microbleeds indicated the increased *K_trans_* in all CSVDs. Fifth, the presence of lacunae or PVS alone was not a good indicator for the increased *K_trans_* in T2DM-related CSVD but can be suitable for non-T2DM CSVDs. The differential correlations between *K_trans_* and each CSVD item of MRI suggested the distinct pathophysiology between T2DM-related CSVD and other CSVDs and supported the high heterogeneity in all CSVDs.

The heterogeneity of CSVD and the dysfunction of BBB were also reported by Zhang et al. Although CSVD is a widespread dysfunction of BBB, the various leakage rates between patients with lacunar stroke and patients with mild vascular cognitive impairment suggested that different pathophysiology exists (24). In their cohort, patients with T2DM accounted for 16% of subjects. Since diabetes-related CSVD is a manifestation of microangiopathy involving the brain (4), this study particularly focused on the effect of T2DM on cerebral permeability. The results also suggested that distinct pathophysiology from other CSVDs exists after eliminating the effects of aging on the breakdown of BBB.

Aging affects BBB integrity in animals (25) and the human brain, particularly in the hippocampus (26). An increase of soluble pericyte marker in the cerebrospinal fluid was found parallel to the BBB leakage, suggesting the critical role of pericyte in maintaining BBB integrity (26). In T2DM, loss of pericyte is an early manifestation of diabetic retinopathy (27). In addition, the normal cellular function of the cerebral pericyte was impaired by T2DM (28). These phenomena possibly resulted in T2DM having an additional negative effect on the maintenance of BBB integrity beyond aging.

In the aspect of T2DM, our results supported the notion that the brain might be involved concurrently with other target organs, as early as the retina and kidneys (4, 29–31). A microvascular leakage in the brain might be similar to the extravasation of the retinal vessels. These target organ damages may share similar mechanisms, by which reactive oxygen species, dysregulation of vascular endothelial growth factor, and other growth factors lead to endothelial dysfunction and subsequently result in damages of filtration barrier and sclerosis (32–34). Increased BBB permeability enables toxic molecules to enter the brain parenchyma, thus initiating multiple pathways of neurodegeneration (35). Subsequently, cognitive decline can develop. In our subjects, a higher prevalence of dementia was found in the T2DM with CSVD group than in the non-CSVD group, supporting the epidemiological observation of the increased risk of cognitive decline in the progression of diabetes (6, 36).

Although the dysfunction of BBB is of generalized nature (24), spatial differences exist. In patients with T2DM, the dysfunction of the BBB in the GM occurred later than that in the WM. This finding is compatible with evolutionary biology concepts: the deep GM that governs instinctive behaviors is usually more protected and less vulnerable to injuries than the WM. In our study, although the three ROIs present in the deep GM had distinct blood supply, all showed similar temporal trends of permeability in patients with advanced T2DM. However, the two ROIs in the WM, namely the frontal and parietal WM, had varying degrees of BBB dysfunction. The parietal WM was affected more than the frontal WM. Although differences in vascular autonomic innervation and myogenic responses between anterior and posterior cerebral circulation have been reported (37–39), additional studies were required to confirm the primary mechanism.

The design of this study was based on the assumption that the presence of CSVD features on MRI represented a more advanced T2DM than no CSVD features on MRI (23). Although we have set the inclusion criteria as an existing T2DM diagnosis on medical records for at least 5 years, the exact duration of T2DM was hard to clarify in real-world clinical practice. Some parameters can be used to support the chronicity of the group of DM with CSVD, such as higher stages of CKD and higher prevalence of dementia (**Table 1**).

Although all CSVDs shared similar features on conventional MRI, DCE-MRI enabled us to visualize early differences in functions between various subtypes of CSVDs. However, the DCE-MRI protocol was time-consuming and highly dependent on patient cooperation. Any motion artifact compromised the accuracy of measurements. Sedative drugs were not used in this study, considering the uncertainty of their effects on BBB permeability. Therefore, patients with moderate to severe dementia were not examined. In addition, patients with advanced CKD, which represented a more advanced diabetes status, were excluded from the DCE-MRI examination considering the risk of gadolinium nephrotoxicity. These limited the generality of the results.

In brain images with too many lacunae or PVSs, the estimation of the parenchymal intensity was difficult because no homogeneous ROI without any fluid space could be observed. Thus, these patients were excluded from our analysis. To overcome the limitations above and broaden the applicability of cerebral permeability using current commonly available methods for patients who cannot undergo DCE-MRI, CSVD items of conventional MRI may help physicians assess the BBB integrity.

In conclusion, this study demonstrated that the increase of cerebral permeability occurred before the image features of CSVD could be visualized on conventional MRI. The increased permeability was associated with long-lasting disease course rather than recent glycemic control. When DCE-MRI is not available, the commonly applicable CSVD features on conventional MRI, such as microbleeds and moderate WMH, can indicate the increased permeability. Cerebral vascular permeability in T2DM and non-T2DM patients with CSVD correlated with different CSVD items, suggesting distinct pathogenesis for diabetic cerebral microangiopathy.

## Data Availability Statement

The raw data supporting the conclusions of this article will be made available by the authors, without undue reservation.

## Ethics Statement

The studies involving human participants were reviewed and approved by IRB in National Cheng Kung University Hospital. The patients/participants provided their written informed consent to participate in this study.

## Author Contributions

Design and conceptualization of the study: Y-TS and Y-CC. Acquisition and analysis of the data: Y-CC and B-ZL. Recruitment of the subjects: Y-TS. Interpretation of the data: Y-CC and Y-TS. Drafting and revising the manuscript: Y-CC, B-ZL, and Y-TS. Visual analysis of the MR images: Y-CC and Y-TS. Processing the MR images and analysis of the data: Y-CC, B-ZL, and Y-CS. All authors contributed to the article and approved the submitted version.

## Funding

The Ministry of Science and Technology, Taiwan (MOST 109-2635-B-006-003 and MOST 109-2115-M-006-018-MY2) and National Cheng Kung University Hospital (NCKUH-11004035) supported this study.

## Conflict of Interest

The authors declare that the research was conducted in the absence of any commercial or financial relationships that could be construed as a potential conflict of interest.

## Publisher’s Note

All claims expressed in this article are solely those of the authors and do not necessarily represent those of their affiliated organizations, or those of the publisher, the editors and the reviewers. Any product that may be evaluated in this article, or claim that may be made by its manufacturer, is not guaranteed or endorsed by the publisher.

## Abbreviations

CSVD, Cerebral small vessel disease; BBB, blood–brain barrier; T2DM, type 2 diabetes mellitus; DCE-MRI, dynamic contrast-enhanced MRI; IRB, Institutional Review Board; CKD, chronic kidney disease; T1WI, T1-weighted images; FLAIR, fluid-attenuated inversion recovery; T1-FFE, T1-weighted fast-field echo; Gd-DOTA, gadoterate meglumine; WM, white matter; GM, gray matter; ROI, regions of interest; CH, caudate head; PVS, perivascular space; WMH, white matter hyperintensity.
